# Ergosterol Modulates Physicochemical Properties and Conformational Changes in High-Moisture Soy-Wheat Protein Extrudates

**DOI:** 10.3390/foods14213627

**Published:** 2025-10-24

**Authors:** Yang Gao, Song Yan, Kaixin Chen, Qing Chen, Bo Li, Jialei Li

**Affiliations:** Food Processing Research Institute, Heilongjiang Academy of Agricultural Sciences, Harbin 150086, China; gao20031026@163.com (Y.G.); 1261456355@163.com (S.Y.); 4228041@163.com (K.C.); chenqingchen163@163.com (Q.C.); blnky@163.com (B.L.)

**Keywords:** soy protein isolate, wheat gluten, ergosterol, high-moisture extrusion, physicochemical properties, conformational changes, fiber formation

## Abstract

This work explores the impact of ergosterol (ERG) addition (0%, 0.5%, 1.0%, 1.5%, and 2.0%) on the physicochemical properties, conformational changes, and digestive characteristics of soy protein isolate (SPI) and wheat gluten (WG) processed by high-moisture extrusion. The results demonstrated that the incorporation of ERG significantly reduced the apparent viscosity and dynamic moduli of the feedstock system, enhancing melt fluidity and consequently reducing extrusion torque, die pressure, and specific mechanical energy. An appropriate amount of ERG (1.0%) effectively facilitated the development of a distinct fibrous morphology, increased the fibrous degree, lightened the color, and softened the texture. However, excessive addition weakened the fibrous structure due to excessively high fluidity. ERG influenced protein aggregation behavior through hydrophobic interactions, reduced thermal stability, and induced a transition in secondary structure from β-turns to α-helices. The in vitro digestibility initially decreased and then increased, with the lowest value observed at 1.0% ERG. This study indicates that ERG can elevate the performance and value of extruded products by modulating protein structure and rheological behavior, providing a theoretical basis for its application in plant-based meat analogue products.

## 1. Introduction

The surge in global meat demand driven by continuous population growth is exacerbating resource scarcity, emissions of greenhouse gases, strain on land and water systems, as well as posing public health challenges such as zoonotic diseases and microbial safety issues [1,2,3]. This pressing reality, coupled with consumers’ growing pursuit of health-promoting and planet-friendly food products, has collectively driven the rapid development of plant protein-based meat analogues. These analogues aim to recreate the characteristic fibrous network, flavor, and nutritional profile of conventional meat through physical transformation, serving as a key alternative to mitigate the environmental burden associated with conventional animal husbandry. Among various processing technologies, the utilization of high-moisture extrusion (HME) for fabricating plant-based meat analogues has witnessed a significant surge due to its efficient and stable processability, as well as its ability to effectively reconstruct protein structures into meat-like fibrous textures [4]. Under the combined effects of high temperature, high pressure, and intense shear forces during high-moisture extrusion (>40% moisture), plant proteins undergo denaturation, dissociation, and subsequent reorganization. This process promotes their alignment into a fibrous, layered structure that mimics the texture and juicy mouthfeel of conventional muscle tissue [5].

Currently, soy protein isolate (SPI) holds a dominant position as the primary raw material for producing plant-based meat analogues because of its optimal essential amino acid profile, excellent gelling properties, high nutritional value, and cost advantages [6]. Wheat gluten (WG), composed of glutenin and gliadin, possesses outstanding extensibility and elasticity, which is capable of forming a viscoelastic matrix upon hydration [7]. It is frequently employed as a crucial cross-linking agent in synergy with SPI, forming the most widely used raw material combination. However, existing plant-based meat analogues still face challenges in fully replicating the comprehensive sensory experience of animal meat. To address rising consumer sensory expectations, food additives have become a key strategy for enhancing texture, juiciness, tenderness, and flavor in meat analogues. Current research on high-moisture extruded plant-based meat analogues primarily focused on traditional additives, such as polysaccharides [8], enzymes [9], lipids [10], and amino acids [11]. Nevertheless, exploring novel, natural additives capable of modulating protein network structures through different mechanisms is of significant importance for the refined textural design and differentiated innovation of high-moisture plant-based meat analogues. Ergosterol (ERG) is a key fungal metabolite and the major sterol compound within the fungal kingdom, notably abundant in edible mushrooms. As a natural phytosterol, it possesses significant nutritional value and pharmacological activity [12]. Its unique rigid cyclic structure and amphiphilic molecular characteristics [13] enable it to function as a crucial component in biological membranes and molecular interactions [14]. Although existing studies have not yet explored its utilization in the domain of plant-based meat analogues, its potential for interacting with proteins presents a novel and promising avenue for improving high-moisture extruded plant-based meat analogues.

This study provides the first systematic evaluation of ergosterol as a structural modulator in high-moisture extruded plant-based meat analogues. The aim of this study was to investigate investigated the effects of different addition levels (0%, 0.5%, 1.0%, 1.5% and 2.0%) of ergosterol on the physicochemical characteristics of these analogues, including the rheological behavior of the initial material blend, as well as the textural properties, color characteristics, macroscopic and microscopic structures, water-holding capacity (WHC), and oil-holding capacity (OHC) of the meat analogues. Furthermore, through analyses of thermodynamic properties, intermolecular interactions, protein secondary and tertiary structures, and protein digestibility, this research systematically examined how different ergosterol incorporation levels influenced protein structural changes within high-moisture extruded plant-based meat analogues. The findings provide a theoretical basis for the utilization of ergosterol in the development of high-moisture extruded plant-based meat analogue products.

## 2. Materials and Methods

### 2.1. Materials

SPI was supplied from Hagaoke Soybean Food Co., Ltd. (Harbin, China). WG was provided by Qufeng Food Technology Co., Ltd. (Weifang, China). The protein contents of SPI and WG were 91.24% and 80.67%, respectively, by the Kjeldahl method. Ergosterol was sourced from Xi’an Xuquan Biotechnology Co., Ltd. (Xi’an, China). Additional key reagents were purchased from Shangbao Biotechnology Co., Ltd. (Shanghai, China). All reagents and chemicals employed in this study were of analytical grade or higher.

### 2.2. High-Moisture Extrusion Process

Based on preliminary experiments, high-moisture extruded plant-based meat analogues were prepared using an SPI: WG mass ratio of 7:3 (yielding optimal texture properties). The extrusion feedstock was formulated by blending the SPI-WG mixture with ergosterol (ERG) at the following mass ratios (*w*/*w*): 100:0 (0% ERG), 99.5:0.5 (0.5% ERG), 99:1 (1% ERG), 98.5:1.5 (1.5% ERG), and 98:2 (2% ERG). High-moisture extrusion of plant-based meat analogues was conducted using a co-rotating twin-screw extruder (CLEXTRAL EV-25, Clestero Co., Ltd., Firminy, France) (L/D ratio 24:1, screw diameter 25 mm, barrel length 600 mm) outfitted with a cylindrical cooling die (80 × 300 mm, D × L). The barrel comprised six independently temperature-controlled zones set to 30, 60, 90, 120, 140, and 140 °C from feed to extruder end.

Operational parameters were maintained as follows:

Screw speed is 300 rpm;

Dry feed rate is 2 kg/h;

Water injection rate is 3 kg/h;

Die temperature is 30 °C.

After achieving steady-state extrusion, samples were collected and labeled as ERG-0 (control), ERG-0.5, ERG-1.0, ERG-1.5, and ERG-2.0 according to ergosterol incorporation levels. All meat analogues were vacuum-sealed immediately post-collection. A part of the freshly prepared samples was stored at 4 °C and analyzed within 24 h, and the residual samples were subjected to freeze-drying and then ground to a fine powder using a 100 mesh sieve and stored at −20 °C for subsequent compositional characterization.

### 2.3. Rheological Measurements

A base mixture of SPI and WG in proportions of 70% and 30% by weight was blended with ERG at varying concentrations (0%, 0.5%, 1.0%, 1.5%, and 2.0%, *w*/*w*). The resulting blend (5 g) was then added to deionized water (20 mL) and magnetically stirred at 1200 rpm for 3 h by a magnetic stirrer (MSP-10C, Kexing Instrument Co., Ltd., Shanghai, China). The resulting blend (5 g) was then added to deionized water (20 mL) and stirred at 1200 rpm for 3 h. The mixture was subsequently equilibrated under 4 °C conditions for 24 h to ensure complete hydration. Rheological properties were determined with a rheometer (Discovery HR-2, TA Instruments, New Castle, DE, USA), following a method adapted with slight modifications from Chen et al. [15]. Measurements employed a 40 mm parallel plate. Before analysis, the sample was allowed to equilibrate for 5 min at 25 °C, and uniformly loaded onto the plate, maintaining a 1 mm measuring gap set in the parallel-plate geometry. Apparent viscosity was measured by a continuous shear rate sweep from 1 to 100 s^−1^, with data points collected across the entire range, at a constant temperature of 25 °C under a 1% constant strain. Dynamic viscoelastic properties were determined by performing frequency sweeps over an angular frequency range of spanning 0.1 to 100 under isothermal conditions (25 °C). The key rheological parameters, namely the storage modulus (G′), loss modulus (G″), and loss tangent (tan δ = G″/G′), were determined. During the thermal sweep, the temperature was first gradually ramped from 30 °C to 110 °C at a heating rate of 10 °C/min and held constant at 110 °C for 1 min, then cooled at a rate of 10 °C/min to 30 °C. Each sample formulation was tested in 3 replicate measurements.

### 2.4. Extrusion Response Parameters

The key extrusion parameters, including torque (N·m), die temperature (°C), and die pressure (bar), were monitored in real-time using an online system. When the extruder operates steadily, measure and record the weight of the extrudate obtained from the cooling die over a one-minute period, which was the extrudate output (g/min). The output was the mass flow rate (MFR) of SPI-WG meat analogues. We computed the Specific Mechanical Energy (SME) according to the following equation:

(1) $$SME\;\left(kJ/kg\right)=\frac{2\pi \times n\times T}{MFR}$$

SME = Specific Mechanical Energy, n = Screw speed (rpm), T = Torque (N·m), and MFR = Mass flow rate (g/min).

### 2.5. Color Properties

The brightness (L*), redness (a*), and yellow (b*) of the meat analogues were determined with a handheld colorimeter (CR-400, Konica Minolta, Tokyo, Japan). Each sample formulation was tested in 5 replicate measurements. The browning index (BI) was derived from the measured L, a, and b* values according to the following equation:

(2) $$BI=\frac{100}{0.17}\times \left(\frac{a^{*}+1.75\times L^{*}}{5.645\times L^{*}+a^{*}-3.012\times b^{*}}-0.31\right)$$

### 2.6. Texture Profile Analysis (TPA)

The hardness, springiness, and chewiness of meat analogues were measured using a texture analyzer (TA-XT Plus model, Stable Micro Systems, Godalming, Surrey, UK) configured for Texture Profile Analysis (TPA) mode. The method was adapted with minor modifications from Gao et al. [16]. Fresh samples were prepared as 10 × 10 × 4 mm cuboids and attached to the instrument base. The samples underwent two sequential compression cycles at 75% deformation of their original height, employing a P/36R cylindrical probe. The experimental parameters are set as follows: the pretest and posttest rates are 2 mm/s, the compression rate is 1 mm/s. Each sample formulation was tested in 5 replicate measurements.

### 2.7. Fibrous Degree

According to the slightly adjusted experimental procedure adopted by Peng et al. [11], the newly prepared extrusion was cut into a block sample with a size of 15 × 15 × 4 mm, placed on a texture instrument test platform, and its fibrous degree was determined by using an HDP/BS probe. The experimental parameters are set as follows: the pretest and posttest rates are 2 mm/s, the compression rate is 1 mm/s, and the compression deformation amplitude is 75%. Each group of samples was measured 5 times.

### 2.8. Water Holding Capacity (WHC) and Oil Holding Capacity (OHC)

Disperse 1 g (W_1_) of extrusion freeze-dried powder in deionized water (20 mL), transferred to a centrifuge tube and vortexed. Centrifuge at 4500 rpm for 20 min, then pour off the supernatant. Invert the tube and obtain the sample weight (W_2_) [11]. OHC can be determined by adding 20 mL of soybean oil. Each sample formulation was tested in 3 replicate measurements. We computed the WHC and OHC according to the following equations:

(3) $$WHC/OHC=\frac{W_{2}}{W_{1}}\times 100\%$$

### 2.9. Microstructure Observation

Fixation of the meat analogues (3 × 3 × 2 mm) was carried out using a glutaraldehyde solution (pH 6.8) for 4 h. Following fixation, dehydration of the samples was carried out by rinsing with a graded ethanol series. They were then thoroughly washed with a tert-butanol/ethanol mixture (1:1, *v*/*v*). We imaged the microstructure of the meat analogues with a scanning electron microscope (SEM) (s3400N, Hitachi, Tokyo, Japan). Observations were performed at magnification levels of 1500×.

### 2.10. Particle Size Distribution (PSD)

Based on the previous method [16], a 1 mg/mL solution was prepared by dispersing the lyophilized powder in 0.02 M phosphate-buffered saline (PBS, pH 7.0) for particle size analysis. Using a particle size analyzer (Mastersizer 3000, Malvern Panalytical Instruments, Malvern, UK), the measurements were performed in triplicate under ambient conditions (25 °C). The apparatus was set to a measurable range of 0.01–3500 μm, with the refractive index for protein fixed at 1.145. Each sample formulation was tested in 3 replicate measurements.

### 2.11. Thermal Properties

The fresh meat analogues (5–8 mg) were loaded into aluminum pans and hermetically sealed for differential scanning calorimetry (DSC) (Q2000, TA Instruments, New Castle, DE, USA) analysis. They were then heated from 20 °C to 220 °C at a rate of 10 °C/min under a nitrogen gas purge with a constant flow rate of 50 mL/min.

### 2.12. Fourier Transformed Infrared Spectroscopy (FTIR)

Sample powder (1 mg) was combined with 100 mg of KBr, thoroughly homogenized by grinding, and compressed into pellets following the procedure of Peng et al. [11]. Spectra were scanned using a Fourier transform infrared spectrometer (Nicolet iS50, Thermo Fisher Scientific, Waltham, MA, USA) in the range of 4000–400 cm^−1^ with a resolution of 4 cm^−1^, averaging 32 scans per spectrum. To investigate secondary structural changes in proteins, Fourier self-deconvolution analysis was performed on the amide I band (1700–1600 cm^−1^) using PeakFit software (PeakFit 4.12, SeaSolve Software Inc., San Jose, CA, USA). Each sample formulation was tested in 3 replicate measurements.

### 2.13. Intrinsic Fluorescence Spectroscopy

According to Chen et al. [17], preparation of the 1% (*w*/*v*) sample solution in 0.1 M PBS (pH 7.0) involved magnetic stirring for 1 h and subsequent centrifugation at 10,000 rpm for 15 min. The supernatant was collected, diluted to 0.4 mg/mL, and its fluorescence intensity was measured with a fluorescence spectrophotometer (F-4700, Hita-chi High-Tech, Tokyo, Japan). With the excitation wavelength fixed at 290 nm, the emission spectrum was recorded from 300 to 500 nm (slit width = 5 nm for both excitation and emission monochromators). Each sample formulation was tested in 3 replicate measurements.

### 2.14. In Vitro Protein Digestibility

In vitro digestion was performed following the standardized INFOGEST static protocol, with some modifications [18]. The process involved successive oral, gastric, and intestinal digestive phases. For the oral phase of digestion, 3 g of samples were blended with 3 g simulated salivary fluid (pH 7, electrolytes, and 75 U/mL α-amylase) and subjected to a 3 min incubation at 37 °C under continuous stirring. The gastric digestive phase was then initiated by adding simulated gastric fluid (pH 3, electrolytes, 2000 U/mL pepsin, and 60 U/mL gastric lipase) to the obtained oral digesta to achieve a ratio of 1:1 (*vol*/*vol*) followed by incubation under constant stirring at 37 °C for 2 h. The intestinal phase was initiated by adding an equal volume of simulated intestinal fluid (pH 7; electrolytes, 100 U/mL pancreatin, bile, and NaHCO_3_) to the gastric digesta. The mixtures were then incubated for an additional 2 h at 37 °C with constant agitation. Subsequently, the mixtures were subjected to a 5 min heat treatment at 90 °C to make enzymes inactivated. Following natural cooling to ambient temperature, the resulting mixtures were harvested as the final digesta. The digesta was distributed among centrifuge tubes and stored at −80 °C for the further analysis.

Protein digestibility was evaluated by trichloroacetic acid (TCA) precipitation, as described by Wang et al. [19]. The digesta was treated with an isovolumetric amount of 15% aqueous TCA solution. After 30 min of incubation, the mixture was vacuum-filtered. Nitrogen content determination was performed using the Kjeldahl method on the filtrate residue and the undigested sample. Each sample formulation was tested in 3 replicate measurements. Protein digestibility was subsequently calculated as follows:

(4) $$Protein\;digestibility\;(\%)=(\frac{N_{0}-N_{1}}{N_{0}})\times 100$$

where N_0_ is the total nitrogen content of the undigested sample, and N_1_ is the nitrogen content of the filtrate residue obtained following TCA precipitation and filtration.

### 2.15. Statistical Analysis

All measurements were repeated independently at least three times. Data are expressed as mean ± standard deviation (SD). The effect of ergosterol addition level was analyzed using a one-way analysis of variance (ANOVA). Prior to ANOVA, the assumptions of normality and homogeneity of variances were verified. Where a significant F-value was obtained (*p* < 0.05), Tukey’s post hoc test was applied to compare means. All statistical analyses were performed using SPSS software (Version 19.0, IBM Corp., Armonk, NY, USA), and a *p*-value of less than 0.05 was considered statistically significant.

## 3. Results and Discussion

### 3.1. Rheological Properties Analysis

**Figure 1:**
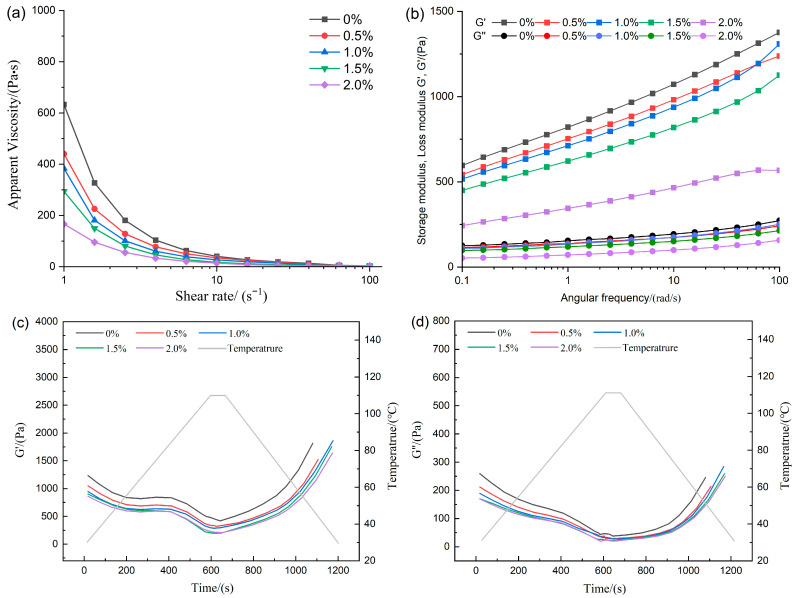
The effect of ERG addition on rheological properties. (**a**) Apparent viscosity. (**b**) Frequency sweep. (**c**,**d**) Temperature sweep.

The evaluation of the rheological behavior provided critical indicators for analyzing the flow properties and viscous characteristics of the feedstock system, thereby revealing the intrinsic relationships among the interactions of various components [20]. As shown in Figure 1a, all formulations displayed pronounced shear-thinning, indicating that the samples were non-Newtonian fluids and that a decline in apparent viscosity was observed with increasing shear rate [10]. The incorporation of ERG led to a marked reduction in the apparent viscosity of the feedstock system. This suggests that as the amount of ERG increases, the viscosity of the system decreased. The reason lies in the fact that the hydroxyl moiety in ergosterol imparts hydrophilic character to the molecule, meanwhile the sterol ring structure contributes hydrophobicity, endowing it with significant emulsifying properties [21]. During high-moisture extrusion, when water was added to the raw material mixture, the amphiphilic nature of ERG [22] allowed it to adsorb at the interface between water and the raw materials, resulting in a more emulsified system. This system was uniformly dispersed, thereby exhibiting high lubrication properties [23], which led to a drop in the viscosity of the base material as ERG increases. The effect of ERG on the viscoelasticity of the feedstock system was further investigated via performing oscillatory frequency sweeps. The measured data of the storage modulus (G′) and loss modulus (G″) are presented in Figure 1b. G′ and G″ represent elasticity and viscosity, respectively. Both the storage modulus (G′) and loss modulus (G″) exhibited a corresponding increase with rising frequency, indicating the gradual unfolding of protein molecules during oscillation. Moreover, throughout the tested frequency spectrum, the storage modulus (G′) consistently exceeded the loss modulus (G″), demonstrating that the feedstock system exhibits viscoelastic gel-like behavior with dominance of elastic properties [15]. The incremental addition of ERG progressively reduced the magnitude of both G′ and G″, in agreement with the observed apparent viscosity data. The reason was that the addition of ERG provided a lubricating effect, leading to a reduction in G′ and G″ as the ERG content in the feedstock system increased, thereby decreasing the viscoelasticity of the system. This resulted in a marked improvement in the flow properties of the base material during the subsequent extrusion process.

Temperature sweep tests were conducted on the feedstock system to predict the evolution of the storage modulus (G′) and loss modulus (G″) during high-moisture extrusion, particularly in response to temperature variations. The morphological development of high-moisture extrudates is governed by the rheological behavior of proteins during cooling [24]. As shown in Figure 1c,d, the storage modulus (G′) and loss modulus (G″) of the tested samples decreased as the temperature increased from 30 °C to 110 °C. This decline may be attributed to the breakdown of the protein network’s integrity in the raw material mixture under the high temperature, high pressure, and intense shear conditions characteristic of high-moisture extrusion [25]. As presented in Figure 1c,d, the storage modulus (G′) and loss modulus (G″) of all samples decreased as the temperature increased from 30 °C to 110 °C. The observed reduction arises as a consequence of the progressive breakdown of the network architecture of proteins among the blend constituents under the high temperature, high pressure, and intense shear conditions characteristic of high-moisture extrusion [4]. Subsequently, upon cooling from 110 to 30 °C, a concurrent rapid rise in G′ and G″ was observed. During the cooling phase, cross-linking occurred among the unfolded protein molecules from the extrusion cooking process, leading to the formation of a stronger gel structure [4], which consequently enhanced the magnitudes of G′ and G″. Based on previous apparent viscosity measurements, a negative correlation was observed between the system’s apparent viscosity and its ERG concentration. It is hypothesized that a protein melt with lower viscosity exhibits higher flow rates, thereby affecting the binding capacity between protein molecules and resulting in reduced G′ and G″. These findings indicate that the addition of ERG can alter the rheological properties of the feedstock system, consequently influencing the physicochemical properties of the extrudates.

### 3.2. Extrusion Response Parameters Analysis

**Figure 2:**
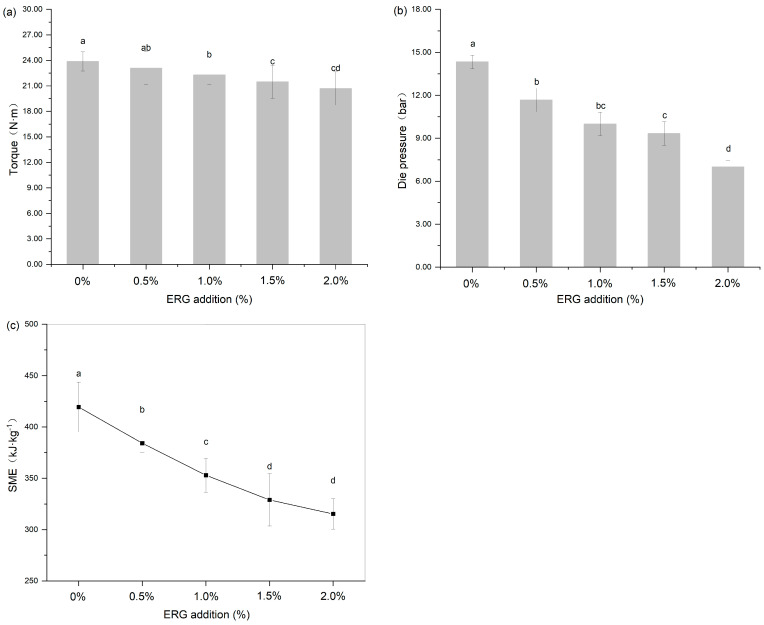
The effect of ERG addition on extrusion response parameters: (**a**) torque (N·m), (**b**) die pressure (bar) and (**c**) specific mechanical energy (SME). Different letters indicate significantly different (*p* < 0.05) as determined by one-way ANOVA with Tukey’s post hoc test.

During high-moisture extrusion, the process conditions are reflected in primary responses such as torque, die pressure, and SME. These parameters directly reflect the processing state and directly reflect the rheological state of the polymer melt in the extruder barrel [26]. They are influenced by raw material characteristics, extrusion processing parameters (such as screw speed, moisture content, and barrel temperature), and equipment parameters (including screw configuration and cooling die dimensions), and are served as determinants for the end-product quality of the extrudates [27]. As shown in Figure 2, the addition of ERG significantly (*p* < 0.05) affected the above extrusion response parameters, including screw torque, die pressure, and SME (calculated using Equation (1)). As the ERG addition level increased from 0% to 2.0%, screw torque, die pressure, and SME exhibited a decreasing trend. This indicates that the incorporation of ERG into the SPI-WG mixture significantly reduced the screw torque, die pressure, and SME during HME processing. In this study, the extrusion processing parameters (screw speed, moisture content, barrel temperature) and equipment parameters (screw configuration, cooling die dimensions) remained constant. Therefore, the reduction in screw torque, die pressure, and SME can be attributed to the decreased melt viscosity of the protein during extrusion resulting from the addition of ERG. This phenomenon occurs because ergosterol, as a phytosterol, possesses a hydroxyl group in its chemical structure that provides hydrophilicity, while its aliphatic/cyclic structure contributes hydrophobicity. This amphiphilic nature endows ergosterol with potential emulsifying properties, and its addition can alter the aggregation state and rheology of proteins [22]. Meanwhile, ergosterol reduces interfacial tension in emulsion systems [21], potentially weakening intermolecular interactions between protein chains and leading to a marked decline in the melt viscosity under high-moisture extrusion conditions. These findings indicate that ergosterol can reduce screw torque, die pressure, and SME by weakening the protein network strength and lowering melt viscosity. The significant reduction in these extrusion parameters is of great technological importance, as it indicates that ERG can lower the energy consumption during processing and reduce the mechanical wear and tear on the extruder, leading to potential cost savings in industrial production.

### 3.3. Color Properties Analysis

Color, as a key sensory attribute of meat analogues, significantly influences consumer acceptance and is affected by both Maillard reactions during extrusion and the inherent color of raw materials [28]. The color variations in extrudates following ERG incorporation were assessed by measuring the L, a, b* values and browning index (BI). As shown in Table 1, when no ERG was added, the extrudates exhibited the lowest L value (61.79) and the highest BI value (36.59), indicating the darkest color. With increasing ERG content, the L* value of the extrudates gradually increased, while the BI value decreased, suggesting that the addition of ERG resulted in a smoother surface and lighter color. This can be attributed to the white color of ERG itself, which is lighter than that of the SPI-WG raw material system, thereby influencing the final color of the extrudates. Furthermore, the Maillard reaction is a major factor influencing extrudate color during high-moisture extrusion. This reaction involves the accelerated condensation of sugars and amino acids under high temperatures, leading to the formation of melanoidins that darken the product [29]. The reduced apparent viscosity from ERG addition in high-moisture extrusion facilitated a higher flow rate of the protein melt. These findings indicate that the melt passes through the extrusion cooking zone more rapidly, reducing the amount of thermal energy absorbed and lowering the melt temperature. Consequently, the Maillard reaction was mitigated, leading to lighter-colored extrudates. The ability of active ingredients, such as the ergosterol in this study and polyphenols or eugenol in composite films reported elsewhere, to modulate color parameters by inhibiting Maillard reaction underscores a common mechanism for improving color stability in protein-rich foods [6].

### 3.4. Textural Properties Analysis

The textural properties of meat analogues are closely related to consumer acceptance and are typically evaluated using parameters such as hardness, springiness, chewiness, and fibrous degree [16]. Textural characterization of the extrudates, including hardness, springiness, and chewiness, was performed by texture profile analysis (TPA). As shown in Table 2, with the increase in ERG in the feedstock system, the springiness of the extrudates showed no significant difference, while the hardness and chewiness exhibited a decreasing trend. When no ERG was added, the extrudates displayed the highest hardness and chewiness, measuring 3901.79 g and 3176.04 g, respectively. A progressive reduction in hardness and chewiness was observed with the incremental addition of ERG. The textural softening effect observed in the extrudates is directly linked to the inclusion of ERG. The observed effect arises from the inherent chemical structure of ERG, whose rigid sterol ring system promotes hydrophobic interactions, thereby disrupting the protein matrix [30]. With increasing ERG content in the feedstock system, ERG inhibits the formation of the protein gel network through hydrophobic interactions [31], thereby leading to a reduction in the hardness and chewiness of the extrudates.

Fiber formation is quantitatively evaluated using fibrous degree as a primary metric for structural alignment, and an increase in its value directly reflects the enhancement of fibrous structure [32]. As shown in Table 2, adding an appropriate amount of ERG to the feedstock system increased the fibrous degree of the extrudates. As the ERG content increased (0–2.0%), the fibrosity of the extrudates initially increased before decreasing. When no ERG was added, the extrudates exhibited the lowest fibrous degree (1.20). At an ERG addition level of 1.0%, the extrudates achieved the highest fibrous degree (1.60), indicating that an appropriate amount of ERG promoted the emergence of a fibrous morphology. Based on the rheological results presented in Section 3.1, the addition of ERG provided a lubricating effect, reducing the viscosity of the feedstock system and improving its fluidity. The establishment of a thermal gradient-induced velocity profile during the flow of protein melt through the cooling die serves as a critical driver for the development of fibrous textures in high-moisture extrudates [24]. Therefore, an appropriate addition of ERG improved the fluidity of the feedstock system and promoted the emergence of a fibrous morphology. However, when the ERG addition level exceeded 1.5%, the fibrosity of the extrudates decreased, suggesting that excessive ERG hindered the emergence of a fibrous morphology. The likely reason is that an excessive lubricating effect caused the melt flow rate to become too high, minimizing the dwell period of the melt in the extruder barrel. This reduced the energy absorbed by the material and lowered the melt temperature [33], preventing the complete unfolding of protein molecules. These findings indicate that the proteins were unable to form a continuous network during reaggregation in the cooling die, leading to a decrease in the fibrous degree of the extrudates.

### 3.5. Water Holding Capacity (WHC) and Oil Holding Capacity (OHC) Analysis

**Figure 3:**
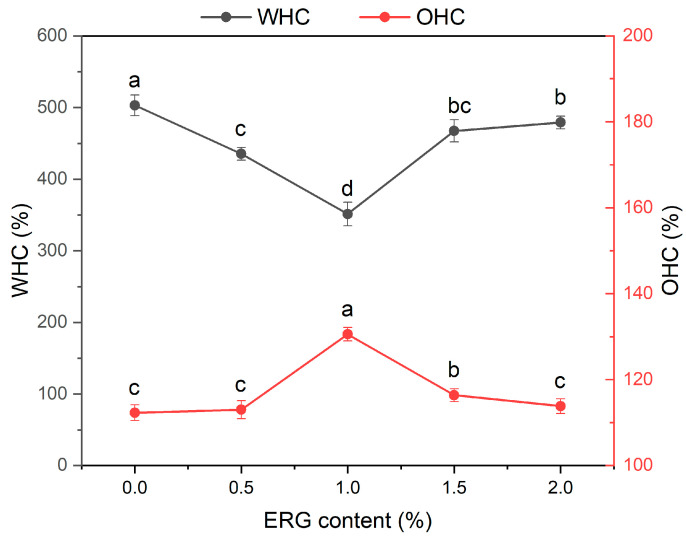
The effect of ERG content on the WHC and OHC of extrudates. Note: Different letters of the same indicator mean significant differences (*p* < 0.05) as determined by one-way ANOVA with Tukey’s post hoc test.

Water-holding capacity (WHC) and oil-holding capacity (OHC) are commonly used to evaluate the water and oil holding capacities of the extrudates, respectively. WHC is governed by multiple factors including protein conformation, the presence of hydrophilic groups and carbohydrates, physical capillary action, and protein primary structure, with its magnitude ultimately dictated by the availability of polar binding sites [34]. OHC is directly dictated by the availability of accessible hydrophobic sites on the material’s surface [29]. As shown in Figure 3, with incremental ERG addition, the water-holding capacity (WHC) of the extrudates progressively declined and then rose, while the oil-holding capacity (OHC) concurrently displayed an inverse trend. When the ERG content increased from 0% to 1.0%, the WHC of the extrudates decreased, while the OHC increased. This may be attributed to the strong hydrophobic interactions associated with ERG [30]. During high-moisture extrusion, the hydrophobic moieties of ERG associate with non-polar amino acid residues on proteins via hydrophobic interactions, thereby diminishing both the degree of protein cross-linking and the population of available water-binding sites [26]. This inhibits the interaction between proteins and water, leading to a decrease in WHC. Meanwhile, the increase in ERG content resulted in a greater number of surface hydrophobic groups, thereby enhancing the OHC of the extrudates. When the ERG content increased from 1.0% to 2.0%, the WHC of the extrudates gradually increased, while the OHC decreased. The observed increase in WHC arises from the amphiphilic nature of ERG [22]. Its molecular structure promotes hydrophilic site formation and water absorption, consequently improving water retention. At the same time, excessive ERG, owing to its strong lubricating effect, reduced the complete application of thermal and mechanical energy on the protein melt [33]. These findings indicate that partial unfolding of the protein molecules was observed, and hydrophobic groups were not completely exposed, decreasing the number of surface hydrophobic groups and ultimately reducing the OHC.

### 3.6. Microstructure and Visual Observation

**Figure 4:**
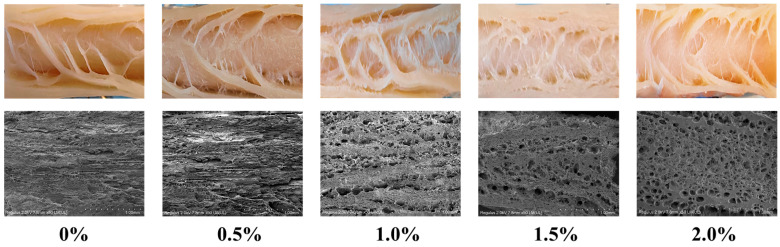
SEM and visual images of extrudates.

Scanning electron microscopy (SEM) provides high-resolution insights into the microstructural features of meat analogues, and when combined with macroscopic structural analysis, it provides comprehensive insights into their fibrous morphology [4]. As presented in Figure 4, macroscopic images of the extrudates demonstrated that without ERG addition, the fibrous structure was not distinctly visible, and a layered morphology was observed. Microscopic examination further indicated that the gel structure formed in the absence of ERG was relatively dense. In contrast, with increasing ERG addition, distinct fibrous structures became apparent in the extrudates. At an ERG addition level of 1.0%, the fibrous structure was most pronounced. However, a progressive increase in ERG content led to a gradual deterioration of the fibrous architecture. Microscopic analysis showed that the gel structure became porous and loose, with less distinct fiber orientation, which is consistent with the earlier measurements of the fibrous degree. These findings demonstrate that an appropriate amount of ERG promotes the emergence of a fibrous morphology in the extrudates. This can be attributed to the notable emulsifying properties of ergosterol [21], which enhance the fluidity of the feedstock system. During high-moisture extrusion, the velocity gradient of the protein melt during cooling is a critical factor for the emergence of a fibrous morphology in the extrudates [24]. These findings indicate that adding an appropriate amount of ERG facilitates the development of this structure. Conversely, excessive ERG addition hinders fiber formation and results in a looser structure. Firstly, the strong lubricating effect leads to an excessively high melt flow rate [33], impairing protein reaggregation in the cooling die. Secondly, the pronounced hydrophobic interactions of ERG inhibit the development of a protein gel network [31], which is unfavorable for the development of a well-oriented fibrous structure along the extrusion direction.

### 3.7. Particle Size Distribution

**Figure 5:**
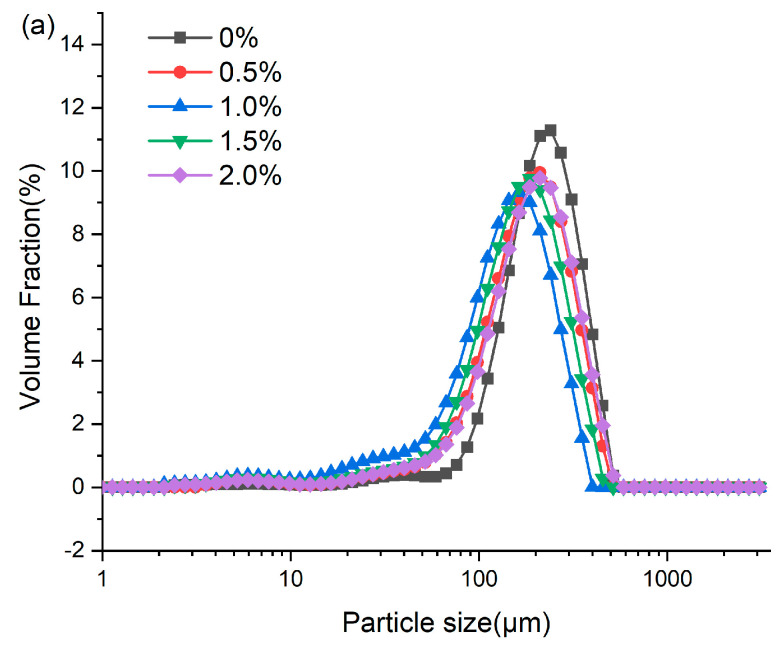

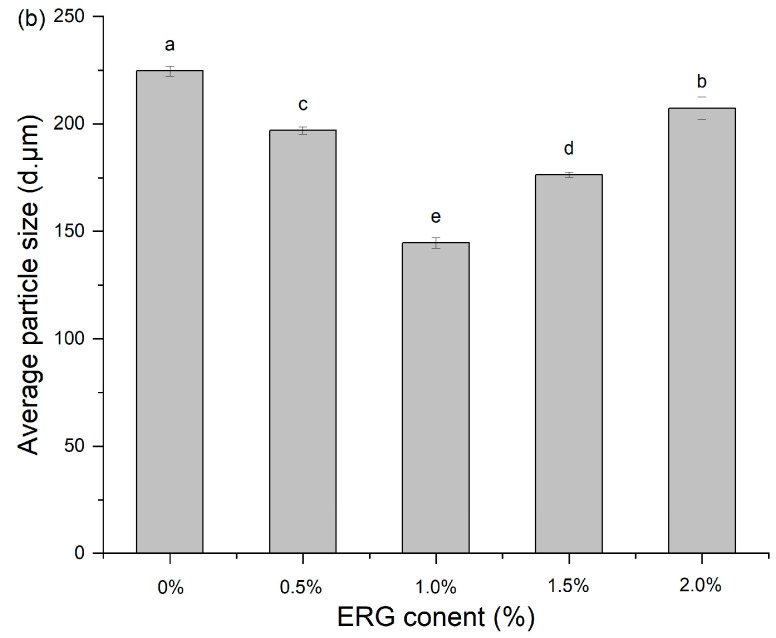
(**a**) Particle size distribution of extrudates. (**b**) Average particle size of extrudates. Note: Different letters of the same indicator mean significant differences (*p* < 0.05) as determined by one-way ANOVA with Tukey’s post hoc test.

Analysis of the extrudate particle size distribution serves as a probe for characterizing protein aggregation during high-moisture extrusion [16]. As presented in Figure 5, the addition of ERG significantly influenced the granulometric profile of the extrudates. All samples exhibited a unimodal distribution, primarily within the range of 10–1000 μm. As the ERG content in the feedstock system increased, the mean particle size of the extrudates declined initially before rising. When the ERG addition level increased from 0% to 1.0%, the experimental results showed a diminution of the extrudates’ mean particle size. The primary reason for this is the strong hydrophobic interaction associated with ERG. As the ERG content increased, it inhibited the development of a protein gel network through hydrophobic interactions [31], thereby impeding protein aggregates of larger size formed and resulting in a reduction in average particle size. Conversely, when the ERG addition level exceeded 1.5%, the average particle size of the extrudates increased with further ERG addition. This phenomenon may be attributed to the amphiphilic nature of ergosterol, which confers emulsifying properties [21]. These findings indicate that the addition of ERG lubricated the feedstock system, reducing its viscosity and improving fluidity. However, excessive ERG, due to its strong lubricating effect, led to an excessively high melt flow rate, minimizing the dwell period of the melt in the extruder barrel. This reduced the energy absorbed by the material and lowered the melt temperature [33], resulting in incomplete protein denaturation during extrusion. Consequently, partial unfolding of the protein molecules was observed and disaggregated, leading to an increase in average particle size.

### 3.8. Thermal Behavior Analysis

**Figure 6:**
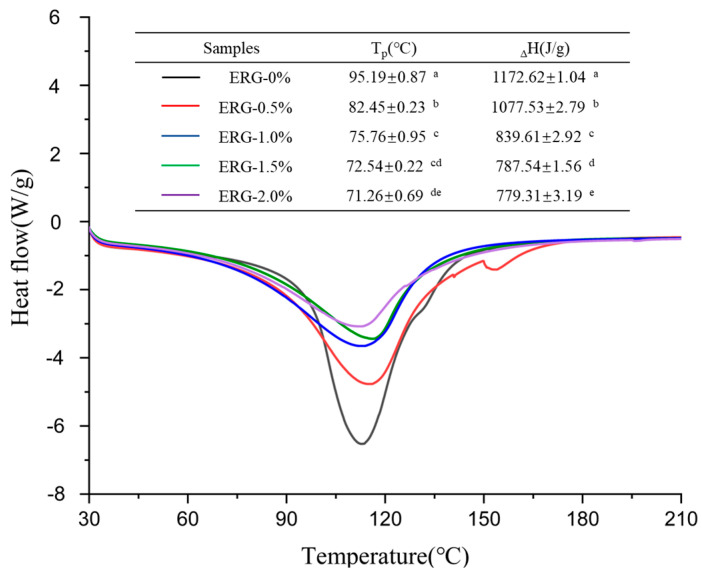
DSC thermograms of extrudates. Note: Different letters of the same indicator mean significant differences (*p* < 0.05) as determined by one-way ANOVA with Tukey’s post hoc test.

During high-moisture extrusion, the extent to which proteins are denatured directly influences the development of fibrous networks. Differential scanning calorimetry (DSC) finds common application in evaluating the denaturation level of proteins and the energy changes associated with protein conformational transitions during thermal processing [26]. The peak temperature (T_p_) serves as an indicator of the thermal stability associated with protein conformational changes, while the enthalpy change (_Δ_H) indicates the extent to which proteins undergo aggregation within high-moisture extrudates [5]. As shown in Figure 6, all extrudates exhibited a major endothermic peak. Both the peak temperature (T_p_) and denaturation enthalpy (_Δ_H) decreased with increasing ERG addition, suggesting that the presence of ERG resulted in structural alterations of the protein and compromised the extrudates’ thermal stability. This phenomenon may be attributed to the strong hydrophobic interactions associated with ERG [30]. As the ERG content in the feedstock system increased, ERG inhibited the formation of the protein gel network through hydrophobic interactions [31]. The new conformational state formed by the binding of ERG to proteins exhibited lower thermal stability compared to the native protein conformation, weakening the stability of the proteins against thermal transformation [35]. Consequently, the extrudates exhibited a drop in thermal stability, leading to reductions in T_p_ and _Δ_H.

### 3.9. Fourier Transformed Infrared Spectroscopy (FTIR) Analysis

**Figure 7:**
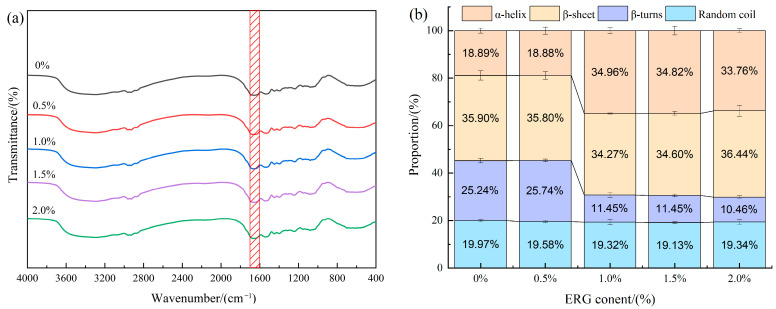
(**a**) FTIR curves for extrudates; (**b**) Secondary structure percentage of extrudates. The red area represents the amide I band with a wavelength range of 1700–1600 cm^−1^.

The FTIR spectra for the various samples are presented in Figure 7a. The amide I band (1700–1600 cm^−1^) is one of the characteristic infrared absorption bands of proteins and serves as a common tool for quantifying protein secondary structures [36]. Specific regions within this band correspond to distinct protein secondary structures: α-helix (1651–1660 cm^−1^), β-sheet (1600–1639 cm^−1^), β-turn (1661–1700 cm^−1^), and random coil (1640–1650 cm^−1^) [37]. As illustrated in Figure 7b, the relative content of secondary structures in the samples was determined by processing this spectral region using PeakFit 4.12 software. In protein secondary structures, α-helix and β-sheet are considered ordered structures, which contrast with the non-ordered arrangements of β-turns and random coils [38]. As shown in Figure 7b, all extrudates exhibited four types of protein secondary structures, predominantly composed of ordered structures (α-helix and β-sheet accounting for over 54% of the total). According to the results, the incorporation of ERG drove a conformational transition from β-turn to α-helix, which consequently increased the overall structural ordering of the protein. This phenomenon may be due to the shared stabilization of both α-helix and β-turn by intramolecular hydrogen bonds [11]. During high-moisture extrusion, mechanical shear forces cause the extension of protein molecular chains. The shorter-range hydrogen bonds in β-turn structures are more susceptible to disruption under such conditions. The incorporation of ergosterol introduces a rigid sterol ring structure, which suppresses random coiling of the protein chains, thereby favoring the establishment of long-range hydrogen bonds. This provides a structural framework conducive to the reconstruction of the α-helix [39]. These findings indicate that protein molecules undergo rearrangement throughout the high-moisture extrusion process, and the incorporation of ERG induces a structural shift in the protein, which β-turn structures transition into α-helices.

### 3.10. Intrinsic Fluorescence Spectroscopy Analysis

**Figure 8:**
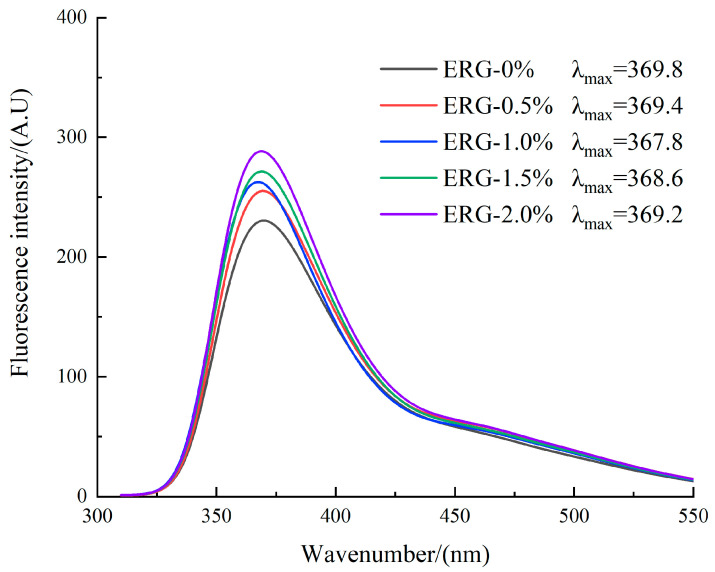
Fluorescence spectra curves for extrudates.

The principle of intrinsic fluorescence spectroscopy relies on its sensitivity to the local microenvironment of tryptophan (Trp) residues, thereby providing an effective means to monitor conformational changes in a protein’s tertiary structure [5]. The local environment of tryptophan can be assessed by its λ_max_: a value less than 330 nm correlates with a hydrophobic region, while a value greater than 330 nm correlates with a hydrophilic region [40]. As presented in Figure 8, the observed λ_max_ values above 330 nm for all extrudates point to hydrophilic microenvironments surrounding the tryptophan residues. The fluorescence intensity gradually increased with higher ERG addition levels. Previous studies have shown that in native proteins, most tryptophan residues are buried within the protein interior. After extrusion processing, protein denaturation leads to the gradual exposure of tryptophan side chains, which typically reduces the fluorescence intensity due to quenching effects from the aqueous environment [41]. This generally results in weaker fluorescence intensity within the protein molecules. With an increase in ERG content from 0% to 1.0%, the fluorescence intensity gradually increased, while the λ_max_ values decreased, indicating a blue shift in the spectrum. This phenomenon arises from the strong hydrophobic interactions conferred by the rigid sterol ring skeleton of ERG [30]. As ERG content increased, the tryptophan residues became surrounded by a hydrophobic environment. The formation of hydrophobic clusters reduced quenching effects [42], leading to a gradual enhancement in fluorescence intensity. Simultaneously, the increase in hydrophobic groups caused the tryptophan residues to reside in an environment of increased hydrophobicity, resulting in a decrease in the emission wavelength of λ_max_. When the ERG content further increased from 1.0% to 2.0%, the fluorescence intensity continued to rise, but the λ_max_ values gradually increased, indicating a red shift. This may be due to the excessive lubricating effect of ERG, which reduced the complete application of thermal and mechanical energy on the protein melt [33]. These findings indicate that the incomplete unfolding and dispersion of protein molecules hindered the exposure of internal tryptophan residues, thereby causing a persistent increase in fluorescence intensity. Meanwhile, because of the amphiphilic nature of ERG [22], its hydrophobic groups participated in the reaggregation of protein molecules, while its hydrophilic groups caused the tryptophan residues to remain in a hydrophilic environment, which caused a red shift of λ_max_.

### 3.11. In Vitro Protein Digestibility

**Figure 9:**
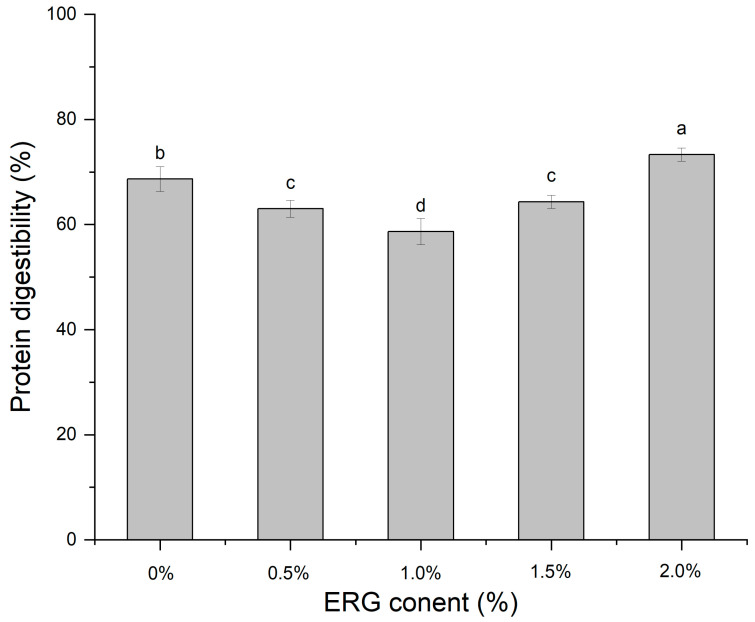
The effect of ERG content on the protein digestibility of extrudates. Note: Different letters of the same indicator mean significant differences (*p* < 0.05) as determined by one-way ANOVA with Tukey’s post hoc test.

The efficiency of protein digestion in vitro directly quantifies the hydrolysis of proteins during gastrointestinal transit, thereby reflecting the impact of ergosterol (ERG) addition levels on the extent of protein digestion [43]. As presented in Figure 9, As the ERG content was raised, the protein digestibility of the extrudates displayed a non-monotonic trend: it initially fell and then climbed. When the ERG addition level increased from 0% to 1.0%, the protein digestibility of the extrudates gradually decreased, and reached a minimum at an ERG content of 1.0%. Which can be explained by the increased ERG content improved the fluidity of the feedstock system, facilitating the development of a fibrous morphology in the extrudates. The well-developed fibrous structure hindered protein digestion, leading to reduced digestibility. However, when the ERG content was further increased from 1.0% to 2.0%, the protein digestibility of the extrudates gradually rose. This can be attributed to the emulsifying effect of ERG, which caused an excessively high flow rate of the protein melt [21], impairing its reaggregation in the cooling die. Simultaneously, the strong hydrophobic interactions of ERG disrupted the structuring of proteins into a gel network [31], exposing more enzymatic cleavage sites and thereby enhancing protein digestibility.

### 3.12. Interrelationship Between Structure, Hydration, and Digestibility

An integrated analysis of the results reveals important correlations between the macroscopic structure, hydration properties, and nutritional functionality of the extrudates. A key observation is the apparent inverse relationship between the fibrous degree and water-holding capacity (WHC) at the optimal ERG concentration (1.0%). While the fibrous structure was most pronounced at this level, the WHC was at its lowest. This can be mechanistically explained by the formation of a highly ordered and dense protein network, where the aligned fibers create a structure with fewer and smaller pores for physically entrapping water compared to the more random, gel-like network in the control sample. Concurrently, this well-developed, dense fibrous structure at 1.0% ERG was associated with the lowest in vitro protein digestibility. It is plausible that the tightly packed and oriented protein filaments physically hindered the access and action of digestive enzymes, thereby reducing the rate and extent of proteolysis. Conversely, when the ERG level was excessive (2.0%), the breakdown of the continuous fibrous network led to a looser, more porous structure. This structural disintegration not only reduced the fibrous degree but also increased the exposure of protein cleavage sites, thereby increasing digestibility, and potentially created larger voids that contributed to the observed slight recovery in WHC. These interrelationships underscore that ergosterol-induced modifications in protein conformation and aggregation fundamentally dictate a trade-off between structural integrity (texture), hydration properties, and nutritional delivery, which is crucial for the tailored design of plant-based meat analogues.

## 4. Conclusions

This study establishes ergosterol (ERG) as a novel, multifunctional natural additive for high-moisture extruded plant-based meat analogues. Its amphiphilic nature enables a dual mechanism of action: serving as an internal lubricant to significantly improve melt fluidity and reduce processing energy, while simultaneously acting as a structural modulator via hydrophobic interactions to control protein aggregation and network formation. An optimal concentration of 1.0% ERG was critical for achieving a superior meat-like structure, maximizing fibrousness and creating lighter-colored, softer-textured meat analogues. Beyond this threshold, excessive lubrication impaired structural integrity. The ERG-induced architecture directly governed nutritional functionality, as evidenced by the modifiable in vitro protein digestibility linked to the density of the fibrous network. Future research should explore the interactions between ergosterol and other biopolymers and the sensory analysis of meat analogues to optimize fiber structure, functional properties, and consumer acceptance as a product in complex food matrices. From an industrial perspective, our findings position ergosterol as a versatile, natural additive that can simultaneously address several key challenges in plant-based meat analogues: reducing processing energy, improving textural quality, enhancing color, and enabling nutritional design. This makes it a highly promising ingredient for the development of superior and sustainable plant-based meat analogues.

## Figures and Tables

**Table 1:** The effect of ERG content on the color properties of extrudates.

ERG Content (%)	L*	a*	b*	Browning Index (BI)
0	61.79 ± 0.41 ^c^	2.26 ± 0.12 ^a^	18.09 ± 0.46 ^c^	36.59 ± 0.64 ^a^
0.5	68.35 ± 0.44 ^b^	0.78 ± 0.05 ^c^	20.21 ± 0.26 ^a^	35.05 ± 0.52 ^b^
1	69.18 ± 0.71 ^a^	1.05 ± 0.04 ^b^	20.20 ± 0.15 ^a^	34.82 ± 0.22 ^b^
1.5	69.79 ± 0.28 ^a^	0.63 ± 0.14 ^c^	19.22 ± 0.28 ^b^	32.11 ± 0.75 ^c^
2	69.59 ± 0.28 ^a^	1.06 ± 0.15 ^b^	19.26 ± 0.35 ^b^	32.79 ± 1.18 ^c^

Note: Different letters in the same column within a same group mean significant difference (*p* < 0.05) as determined by one-way ANOVA with Tukey’s post hoc test.

**Table 2:** The effect of ERG content on the textural properties of extrudates.

ERG Content (%)	Fibrous Degree	Hardness (g)	Springiness	Chewiness (g)
0	1.20 ± 0.02 ^c^	3901.79 ± 248.51 ^a^	0.97 ± 0.02 ^a^	3176.04 ± 260.94 ^a^
0.5	1.54 ± 0.07 ^a^	2549.57 ± 324.26 ^b^	0.94 ± 0.04 ^a^	2120.13 ± 283.72 ^b^
1	1.60 ± 0.03 ^a^	2347.91 ± 78.00 ^bc^	0.96 ± 0.01 ^a^	1887.50 ± 5.81 ^bc^
1.5	1.44 ± 0.03 ^b^	1991.89 ± 370.90 ^c^	0.96 ± 0.09 ^a^	1581.37 ± 134.73 ^c^
2	1.40 ± 0.03 ^b^	1439.46 ± 274.65 ^d^	0.96 ± 0.03 ^a^	1157.00 ± 268.17 ^d^

Note: Different letters in the same column within a same group mean significant difference (*p* < 0.05) as determined by one-way ANOVA with Tukey’s post hoc test.

## Data Availability

The original contributions presented in the study are included in the article. Further inquiries can be directed to the corresponding author.
